# Hypokalemic periodic paralysis as first sign of thyrotoxicosis

**Published:** 2013-03-25

**Authors:** RA Trifanescu, R Danciulescu Miulescu, M Carsote, C Poiana

**Affiliations:** * Department of Endocrinology, “Carol Davila" University of Medicine and Pharmacy, Bucharest, Romania; ** “C.I. Parhon" National Institute of Endocrinology, Bucharest, Romania

**Keywords:** hypokalemic paralysis, thyrotoxicosis, teenagers

## Abstract

Background: periodic paralysis related to hypokalemia is seldom reported in thyrotoxicosis, and it usually occurs in Asian males.

Patients and methods: Two Romanian (Caucasian) young patients presented with hypokalemic paralysis. TSH, FT4, TT3 was measured by immunochemiluminescence.

Case report 1. Patient O.R, aged 19, presented marked asthenia and lower limbs paralysis, following high carbohydrate meal. He declared 10 kg weight loss on hypocaloric diet and mild sweating. Biochemical data revealed moderate hypokalemia (K+=2.6 mmol/L) and thyrotoxicosis (TSH<0.03 mIU/L, FT4=30 pmol/L, TT3=315 ng/dL).

Case report 2. Patient T.A., aged 18, presented 2 episodes of weakness and flaccid paralysis, with hypokalemia, precipitated by effort, without any sign of thyrotoxicosis. Biochemical data revealed severe hypokalemia (K+=1.8 mmol/L) and thyrotoxicosis (TSH<0.03 mIU/L, FT4=24 pmol/L, TT3=190 ng/dL). Treatment with intravenous potassium, thereafter methimazole and propranolol were administered in both cases, with the maintenance of normal kalemia and thyrotoxicosis’ control.

Conclusion: these 2 cases of hypokalemic periodic paralysis occurring in young Caucasian teenagers with mild thyrotoxicosis underlined the importance of thyroid screening in patients with symptomatic hypokalemia, even in the absence of symptoms and signs of thyrotoxicosis.

Abbreviations: THPP=Thyrotoxic periodic paralysis, BMI=body mass index, TRAb=TSH receptor antibody, ECG=electrocardiogram.

## Introduction

Hypokalemia with acute paralysis is a medical emergency. Up to 43.3% of the patients with hypokalemic paralysis had a secondary cause, 16.6-32% showing thyrotoxic paralysis [**1,2**]. Thyrotoxic periodic paralysis (THPP) is an unusual complication of thyrotoxicosis, that manifests as acute episodes of muscle weakness associated with hypokalemia [**3,4**]. The disease primarily affects people of Asian descent, but also other ethnic groups [**5**] such as Europeans [**6**], Hispanics [**7**], native Americans [**8**]. THPP predominantly affects males [3,9]. THPP was reported more frequently in Graves disease [**10**], but also in toxic multinodular goiter [**11**], toxic thyroid adenoma [**12**], TSH secreting pituitary adenomas [**13**], painless thyroiditis [**14**], in patients with Jodbasedow thyrotoxicosis [**15**] and in patients taking levothyroxine [**16**].

## Patients and methods

Two young, non-consanguineous Caucasian patients (18 and 19 years) initially presented with periodic hypokalemic paralysis. They did not have Asian ancestors, or a familial history of periodic paralysis, or personal history of distal renal tubular acidosis, primary hyperaldosteronism or Bartter-like syndrome. Both patients denied diarrhea, diuretics, licorice or alcohol use. 

 TSH, FT4, TT4, TT3 were measured by immunochemiluminescence, upright aldosterone by ELISA, plasma 8 a.m. cortisol and direct rennin by immunochemiluminescence.

## Case reports

Case 1, O.R., male, aged 19, overweight (BMI=27.8 kg/m2), presented with marked asthenia and lower limbs paralysis. The symptoms had abrupt onset in the morning, after a rich carbohydrate meal. The patient declared 10 kg weight loss on hypocaloric diet and mild sweating. Neurological exam showed symmetrical, lower limbs flaccid paresis. Biochemical assessment revealed hyperglycemia (126 mg/dL) and moderate hypokalemia (2.6 mmol/L). Intravenous potassium supplements were administered with normalization of serum potassium levels. In the endocrine department, the physical exam revealed mild tachycardia, small diffuse goiter and tremor, without exophthalmia. Hormonal assessment revealed thyrotoxicosis (TSH<0.03 mIU/L, FT4=30.6 pmol/L, TT3=315 ng/dL); TSH receptor antibodies (TRAb) were positive (**Table 1**). Oral glucose tolerance test was normal. After restoration of normal serum potassium levels, potassium excretion was normal (31.6 mmoL/24 hours). Case 2, T. A., male, aged 18, presented with marked asthenia and quadriplegia with abrupt onset after an intensive physical effort. Neurological exam showed flaccid limbs paralysis. Severe hypokalemia (1.8 mmol/L) was present. Intravenous potassium supplements were administered with normalization of serum potassium levels. In the endocrine department, physical exam revealed small diffuse goiter without tachycardia, tremor or exophthalmia. Hormonal assessment revealed mild thyrotoxicosis (TSH<0.03 mIU/L, FT4=24 pmol/L, TT3=190 ng/dL) (**Table 1**). After the restoration of normal serum potassium levels, renal potassium excretion was normal (51.8 mmoL/24 hours). In both patients, thyroid ultrasound revealed hypoechoic, diffuse goiter; there were no signs of hypercortisolism and adrenal assessment was normal (**Table 1**).

**Table 1:** Clinical and biochemical features at the onset of hypokalemic paralysis

	Case 1	Case 2
Symptoms	Marked asthenia Lower limbs paralysis	Muscle weakness Flaccid quadriplegia
Precipitant factor	Hyperglycaemia (126 mg/dL)	Physical effort
BMI(kg/m2)	27.8	23.2
K+(mmol/L)	2.6	1.8
Na+(mmoL/L)	143	140
TSH(mIU/L)	<0.03	<0.03
FT4(pmol/L)	30.6	24
TT3(ng/dL)	315	190
Thyroid ultrasound	Hypoechoic, diffuse goiter	Diffuse goiter
Plasma 8 a.m. cortisol after 1 mg DXM overnight	0.87	0.57
Aldosterone (ng/dL)/direct renin (ng/L)	0.8	1
Adrenal imaging	Normal ultrasound	Normal CT scan

Antithyroid drugs (Methimazole 30 > 20 > 10 > 5 mg/day) and non-selective β blockers (Propranolol 120 > 60 > 40 > 30 mg/day) treatment were administered in both patients, with long-term maintenance of normal serum potassium levels and progressive normalization of TSH and FT4 levels (**Table 2**). Antithyroid drugs’ treatment duration was of 18 months. No recurrence of the paralysis has been noted during follow-up. 

**Table 2:** Biochemical data after 3 months treatment with antithyroid drugs and non-selective β blockers

	Case 1	Case 2
K+(mmol/L)	4.5	5
TSH(mIU/L)	0.3	0.09
FT4(pmol/L)	14	12.6

To our knowledge, these cases are the first Romanian published cases of hypokalemic thyrotoxic paralysis so far.

## Discussion

Both our patients are males. THPP had a higher predilection for men than for women [**4**]. The disease occurred especially in young adults [**17**]. 

 In our patients, THPP occurred at night and early after awakening, respectively. Early morning paralysis was reported as the first manifestation of hyperthyroidism both in Asian men [**18**] and in Caucasians [**19**]. Precipitating factors included sleep at night, hot weather, upper respiratory tract infections, excessive physical activities [**17**], excessive alcohol intake [**20**], high carbohydrate intake [**21**]. 

 Both our patients showed mild thyrotoxicosis. Many affected patients do not have obvious symptoms and signs of thyrotoxicosis [**3,22**].

 Both patients showed sinus tachycardia, U waves, without ventricular arrhythmia on electrocardiogram (ECG). ECG manifestations in THPP were slightly different as compared with non-thyrotoxic periodic paralysis, at similar low K+ levels: heart rate, PR interval, and QRS voltage were significantly higher [**23**]. Young patients with THPP complicated by acute hypercapnic respiratory failure and ventricular tachycardia [**22**] or with thyroid storm, psychosis and episodic acute respiratory failure [**5**] were reported.

 The underlying mechanism of THPP is an acute extra to intracellular shift of potassium, mainly into the muscles, due to the stimulation of the activity of the Na-K-ATP-ase pump by thyroid hormones, via beta-adrenergic stimulation [**3**]. Genetic predisposition plays a role in the pathogenesis of thyrotoxic hypokalemic periodic paralysis; polymorphisms of the calcium channel alpha1-subunit gene [**24**], mutations in SCN4A or CACNA1S [**25**] were reported. Up to 33% of THPP patients showed a mutation in Kir2.6 gene, regulated by thyroid hormones [**26**]. Single nucleotide polymorphisms of gamma-aminobutyric acid receptor alpha3 subunit were associated with THPP [**27**]. 

 Treatment of THPP implies two steps: the hypokalemia correction and the euthyroidism restoring [**4**]. Both our patients initially received intravenous potassium chloride supplementation; correction of hypokalemia by potassium supplementation, in order to avoid fatal cardiac arrhythmias and to reverse muscle weakness is mandatory [**28,3**]. However, the patients do not have a total body deficiency of potassium, so aggressive potassium supplementation should be avoided, because it can result in rebound hypokalemia [**29**]. Potassium supplementation should be given as small as possible (<10 mmol/hr) [**28**]. Intravenous glucose solution should be avoided, because it could aggravate hypokalemia, leading to death [**30**]. 

 The second step is the treatment with antithyroid drugs and beta-blockers. Nonselective beta-adrenergic blockers can ameliorate and prevent recurrence of the paralytic attacks. Both our cases received propranolol 120 mg/day and antithyroid drugs. 

 In both presented cases, no recurrence of the paralysis has been noted within the first 3 years after diagnosis. However, before achieving the euthyroid status, the rate of recurrent attacks could be up to 62.2% [**17**]. This episodic paralysis will remit with definitive control of hyperthyroidism [**3**]. Both our patients refused definitive surgical treatment of thyrotoxicosis and underwent regular thyroid follow-up.

## Conclusions

Thyrotoxic hypokalemic periodic paralysis, although rare in Caucasian men, should be considered in the differential diagnosis of hypokalemic paralysis. Thyroid screening is mandatory in patients with muscular paralysis associated with hypokalemia.
